# Free school fruit – sustained effect three years later

**DOI:** 10.1186/1479-5868-4-5

**Published:** 2007-02-19

**Authors:** Elling Bere, Marit B Veierød, Øivind Skare, Knut-Inge Klepp

**Affiliations:** 1 Department of Nutrition, Institue of Basic Medical Sciences, University of Oslo, Norway; 2 Department of Public Health, Erasmus University Medical Centre Rotterdam, the Netherlands; 3 Department of Biostatistics, Institue of Basic Medical Sciences, University of Oslo, Norway; 4 Department of Medical Genetics, Ullevål University Hospital, Norway

## Abstract

**Background:**

Norwegian children consume less fruit and vegetables (FV) than recommended. In order to increase the intake, a School Fruit subscription programme is now offered to all Norwegian elementary and junior high schools. This programme has limited effect due to low participation by schools and pupils. However, recent evaluations of the programme offered for free have reported good effects in increasing FV intake. The purpose of the present study is to evaluate the long term effects of the Norwegian School Fruit programme, provided at no-cost to the pupils, three years after it was provided for free.

**Methods:**

A total of 1950 (85%) 6th and 7th grade pupils from 38 Norwegian elementary schools participated in the project. Nine schools were selected as intervention schools and participated for free in the Norwegian School Fruit programme for a school year (October 2001 until June 2002). A baseline questionnaire survey was conducted in September 2001, and follow-up surveys were conducted in May 2002 and May 2005. FV intake was assessed by a written 24-h recall (reporting FV intake at school and FV intake all day), and by four food frequency questions (reporting usual FV intake). Data were analysed by a linear mixed model for repeated measures.

**Results:**

The pupils in the free fruit group increased their FV intake compared to pupils in the control group as a result of the intervention. Some of the effect was sustained three years later. The estimated long-term effects for FV all day were 0.38 and 0.44 portion/day for boys and girls, respectively.

**Conclusion:**

The results show long-term effects of a free school fruit programme.

## Introduction

Norwegian children consume less fruit and vegetables (FV) [1] and more added sugar [1,2] and saturated fat [1], than recommended. A number of intervention studies have demonstrated that it is possible to increase children's FV intake, even though effects have been small and the long-term persistence of such changes are unknown [3]. The more effective studies have been comprehensive multi-component interventions targeting several determinants. However, very few studies have been able to evaluate the effect of the separate components, and little is known about what mediate the effect observed. Studies of single intervention components, targeting specific strong determinants of intake, are largely missing. Recently, an extensive review of determinants of children's and adolescents' fruit and vegetable intake identified availability and accessibility among the strongest determinants [4], a finding also supported by Norwegian studies [5,6]. Thus, an intervention increasing the accessibility of fruit and vegetable should in theory be effective, as is also predicted based on ecological models emphasising the important influence on health-related behaviours exercised by the environment [7].

Most elementary school children in Norway bring their own lunch (usually sandwiches) to school. Few elementary schools have canteens, and traditionally FV have not been available at school. To increase the pupils' intake of FV, a School Fruit Programme is now offered to all Norwegian elementary and junior high schools [8]. Pupils who subscribe receive a piece of fruit or a carrot each school day, usually at lunch time. The cost for the pupils/parents is NOK 2.50 per school day (approximately EUR 0.30). The programme is subsidised by the Norwegian Government by NOK 1.00 per pupil per school day.

A major problem with the current programme is the low participation rate by schools and by pupils at participating schools. Only 12% of the total Norwegian elementary school population (grades 1–10) did subscribe in the spring semester 2006, and the effect on overall FV intake is therefore limited. It is also noticeable that subscribing pupils tend to report healthier behaviours to start with than do non-subscribing pupils [8,9]. Thus, the programme might contribute to increase the inequality seen in FV intake in this age group [9].

This paid programme increases the availability of fruits and vegetables at participating schools. However, it does not increase the accessibility for the individual pupil unless the parents subscribe to the programme on behalf of their children. If the programme was offered for free, it would have increased the accessibility of fruits and vegetables for all children. Recent evaluations of this programme when it was offered at no-cost for the pupils, have shown good effects in increasing all children's FV intake, even among those that usually eat little FV [8]. A sustained elevated FV intake was furthermore found one year after the programme was provided for free [10]. This effect could in part be explained by higher subscription rates in the standard paid Norwegian School Fruit Programme the subsequent year [10]. The no-cost subscription also resulted in decreased consumption of unhealthy snacks, i.e. snacks high in added sugar and/or saturated fat, for pupils of parents without higher education [8].

A recent cost-effectiveness analysis concluded that a School Fruit Programme provided for free to all 10 years of elementary and junior high school would be cost-effective if it resulted in a sustained (lifelong) increase in mean FV intake of 2.5 grams (conservative estimate) per day [11].

The aim of the present paper is to evaluate the effect of the Norwegian School Fruit Programme, provided at no-cost to the pupils, three years after it was provided for free, for both fruit and vegetable intake and consumption of unhealthy snacks.

## Methods

### Design and study sample

A total of 38 randomly drawn elementary schools from two different counties (Hedmark and Telemark) participated in the Fruits and Vegetables Make the Marks project (FVMM) [5]. Nine schools within one of the counties (Hedmark) were randomly selected as intervention schools and participated in the Norwegian School Fruit Programme for free (Free fruit group) during the school year 2001/2002. In the present study the remaining 29 schools served as control schools. The free subscription programme started in October 2001, and lasted throughout the school year (i.e. until June 2002).

A baseline questionnaire survey was conducted in September 2001, while follow-up surveys were conducted in May 2002 and May 2005. In September 2001, all 6th and 7th graders at all schools were invited to participate. In May 2005, the same children were in 9th and 10th grade at 33 junior high schools (most schools were different from the elementary schools). Research clearance was obtained from The Norwegian Social Science Data Services.

The FVMM cohort includes 1950 pupils (participants at baseline): 984 boys and 966 girls, 585 in the Free fruit group and 1365 in the control group. Average age was 11.8 years at baseline. A total of 1794 and 1602 pupils participated at the follow-ups in May 2002 and May 2005 respectively. At baseline 1647 pupils had a parent/guardian who completed a parent questionnaire. The average parental age was 40.0 years, and 84% of the parents were mothers/female guardians.

### Instruments

A survey questionnaire was completed by the pupils in the classroom in the presence of a trained project worker. A written 24-h FV recall was used to assess pupils' FV intake (portions/day). The 24-h recall part of the questionnaire was read aloud to the pupils by a project worker at baseline and at the first follow-up survey, but not at the second follow-up survey as the pupils were older and they could read better. FV intake the previous day was recorded for school days (i.e. the surveys were conducted on weekdays, Tuesday through Friday). FV intake at school and FV intake all day were calculated (portions/day). In addition, usual FV intake was measured by four food frequency questions (times/week), and unhealthy snacks consumption by three food frequency questions (soda/candy/potato chips (crisps)). Both the 24-h recall and the food frequency questions are previously presented, and their validity and reliability have been reported for FV intake among 6th graders [12]. The pupils reported their sex, class level (initial 6th or 7th grade) and whether they subscribed to the school fruit programme or not. Since both the free and the paid conditions are versions of the same programme, this variable is coded yes for both the free and the paid subscription. Parents recorded their level of education at baseline (lower: no college or university education/higher: having attended college or university).

### Statistical analyses

In the study sample, some pupils (66 at baseline, 138 in May 2002 and 134 in May 2005) did not attend school the day before the survey day. Therefore, they were excluded from the analyses of FV at school, but they were included in all other analyses presented.

Data were analysed by a linear mixed model for repeated measures [13], using R software (www.r-project.org). Separate analyses were preformed for each of the four dependent variables; FV at school (portions/day), FV all day (portions/day), usual FV intake (times/week) and soda/candy/chips (times/week). Based on an examination of the residuals, we chose to log_e_-transform all these variables except usual FV intake; back transformed means are presented. The mixed model included both fixed and random effects. As fixed effects, in addition to intervention condition, the models included sex, class level in September 2001 and time (survey). Relevant interaction terms were tested. We also included parents' educational level in the model. However, adding this covariate to the model did not change the results and it did not interact with intervention. Moreover, as parents' educational level was missing for 16 % of the pupils we chose to omit this variable from the statistical models. To adjust for dependency in the measurements, random effects were added for pupil and school, where pupils were nested within schools. Moreover, we allowed for a general correlation structure with time (survey). We were interested in the difference between the intervention and the control groups with respect to the FV and soda/candy/chips intake. At baseline, prior to the intervention, any difference is due to randomness or to selection bias that is assumed independent of the intervention. The intervention effect is defined as the change, from the baseline survey to the respective follow-up surveys, in the difference between intervention group and control group. Finally, we adjusted for individual subscription to the school fruit programme, in May 2002 and May 2005, respectively.

## Results

The third order interaction term between sex, time and intervention was not significant in any of the models for the four response variables (0.13 ≤ p ≤ 0.90). Thus, the intervention effect was similar for boys and girls (on the log_e_-scale, back transformed estimates differ slightly). All analyses included the interaction between time and intervention which represents the intervention effect defined above. This interaction term was highly significant for FV at school, FV all day and usual FV intake (p < 0.001), but not for soda/candy/chips (p = 0.18). In addition, we found a significant interaction between time and sex in the models for FV at school and soda/candy/chips (p-values 0.03 and 0.002, respectively), and a significant interaction between sex and intervention in the models for FV all day and usual FV intake (p-values 0.04 and 0.008, respectively).

The pupils in the Free fruit group significantly increased their FV intake from baseline to May 2002, compared to the control group (Table 1). This short-term effect has previously been described in Bere et al. (2005). Sustained significant effects on FV intake three years after the end of the intervention were also observed (all p-values < 0.001). For boys the estimated change in FV intake from baseline to May 05, compared to the control group, were 0.13 portions/day for FV at school, 0.38 portion/day for FV all day and 1.6 times/week for usual FV intake. Corresponding estimates for girls were 0.15 portions/day, 0.44 portion/day and 1.6 times/week, respectively. No significant intervention effect was observed for soda/candy/chips.

**Table 1:** Effect of free subscription to the Norwegian School Fruit Programme; while provided (May 2002) and three years later (May 2005).

		Adjusted mean values*			Change in adjusted values*	
		Sept. 01	May 02	May 05	Sept. 01 to May 02	Sept. 01 to May 05 (**)
**FV at school (portions/day)**						
Boys	Free fruit (n = 291)	0.16	0.66	0.21	0.50	0.05
	Control (n = 646)	0.19	0.16	0.11	-0.03	-0.09
	difference	-0.03	0.50	0.10	0.53	0.13 (0.07)
	p-value				<0.001	<0.001 (0.04)
						
Girls	Free fruit (n = 273)	0.29	0.76	0.37	0.48	0.08
	Control (n = 644)	0.32	0.24	0.25	-0.08	-0.07
	difference	-0.03	0.53	0.12	0.56	0.15 (0.07)
	p-value				<0.001	<0.001 (0.04)

**FV all day (portions/day)**						
Boys	Free fruit (n = 300)	1.25	1.42	1.22	0.18	-0.03
	Control (n = 670)	1.57	1.18	1.16	-0.39	-0.41
	difference	-0.32	0.24	0.06	0.57	0.38 (0.31)
	p-value				<0.001	<0.001 (0.008)
						
Girls	Free fruit (n = 281)	1.95	2.18	1.91	0.23	-0.04
	Control (n = 675)	2.03	1.57	1.54	-0.46	-0.48
	difference	-0.07	0.62	0.37	0.69	0.44 (0.35)
	p-value				<0.001	<0.001 (0.008)

**Usual FV intake (times/week)**						
Boys	Free fruit (n = 294)	12.1	12.9	12.7	0.8	0.6
	Control (n = 644)	13.2	12.7	12.2	-0.5	-1.0
	difference	-1.1	0.2	0.6	1.3	1.6 (0.9)
	p-value				<0.001	<0.001 (0.04)
						
Girls	Free fruit (n = 273)	15.6	16.4	16.2	0.8	0.6
	Control (n = 651)	15.1	14.6	14.1	-0.5	-1.0
	difference	0.5	1.8	2.2	1.3	1.6 (0.9)
	p-value				<0.001	<0.001 (0.04)

**Soda/candy/chips (times/week)**						
Boys	Free fruit (n = 572)	6.0	5.8	5.9	-0.2	-0.1
	Control (n = 1303)	6.5	6.6	6.6	0.1	0.2
	difference	-0.4	-0.8	-0.7	-0.3	-0.3 (-0.2)
	p-value				0.07	0.31 (0.48)
						
Girls	Free fruit (n = 572)	5.3	5.1	4.6	-0.2	-0.7
	Control (n = 1303)	5.7	5.9	5.2	0.2	-0.6
	difference	-0.4	-0.7	-0.6	-0.3	-0.2 (-0.1)
	p-value				0.07	0.31 (0.48)

* Data were analysed by a linear mixed model for repeated measures. Fixed effects were intervention condition, gender, class level, and time (survey). Random effects were added for pupil and school, where pupils were nested within schools. Interaction effects included in the models: time and intervention condition (all models), sex and intervention condition (FV all day, usual FV intake), time and sex (FV at school, soda/candy/chips).

** Numbers in the parenthesis are adjusted for individual subscription to the school fruit programme. For usual FV intake an interaction between individual subscription and intervention was included.

In May 2002, 100% and 13% of the intervention and control pupils, respectively, subscribed to the School Fruit programme (Table 2). The corresponding numbers were 16% and 1% in May 2005. When subscription to the School Fruit Programme was included in the analyses the effect of the intervention decreased some, and became less significant for all measures, but the effect was still significant for all FV measures (Table 1). We found a significant interaction between individual subscription and intervention for usual FV intake (p = 0.04), but not for FV all day (p = 0.20) and FV at school (p = 0.92).

**Table 2:** Subscription rates in the School Fruit Programme in May 02 and May 05

		School Fruit Programme		
			Pupils subscribing	
Group		Schools participating	% at participating schools (boys, girls)	% of group (boys, girls)
Free fruit	May 02	9 (of 9)	100 (100, 100)	100 (100, 100)
	May 05	2 (of 9*)	31 (26, 35)	16 (13, 19)
Control	May 02	9 (of 29)	44 (43, 46)	13 (13, 14)
	May 05	1 (of 25*)	19 (0, 25)	1 (0, 1)

* One intervention and one control elementary school sent pupils to the same junior high school (total junior high schools in May 05 were 33)

## Discussion

The present results show that free school fruit had a positive effect on Norwegian school children's FV intake, also three years after the fruit was provided for free. Such long term effect of a FV intervention for children has, to our knowledge, not previously been reported. Previously we have reported that a free school fruit programme, in addition to increasing pupil's FV intake, also reduce the consumption of unhealthy snacks among pupil's of parents without higher education [8]. This effect is not sustained three years after the end of the intervention.

The long term effects were estimated to be 0.38 and 0.44 portions/day for FV all day (boys and girls, respectively). A portion in the present study is about 80 grams [12]. The effect of the free fruit intervention three years after the fruit was provided for free can therefore be estimated to be about 30–35 grams/day. This is a considerable larger increase than what is concluded to be needed for the Norwegian School Fruit Programme, offered for free for all 10 years, to be cost effective (2.5 grams as a conservative estimate) – if the effect is sustained throughout life [11]. The observed significant long-term effects in May 2005 decreased some when adjusting for individual subscriptions to the school fruit programme, but the effects were still significant. The subscription rates in May 2005 were clearly higher in the Free fruit group than in the control group, and this then accounted for some of the difference between the groups. Available FV (e.g. such as a paid subscription programme) therefore promotes a sustained long term effect. Longer term follow-up surveys are, however, needed to state whether lifelong FV habits have been established in order to state the cost-effectiveness of the programme.

The long-term effect seen in the present paper contradicts with findings reported from the British National School Fruit Scheme where short-term but no long-term effect was seen [14]. That study had some clear limitations as it was cross-sectional (no baseline data), had a low response rate (51%) and schools were not randomly assigned to condition [14]. In addition, no paid programme (or something equivalent) was offered to the pupils after the intervention. Thus, the setting was different, and it is therefore difficult to compare the results with those from the present study. Different versions of free school fruit initiatives have also been tried out, or are planned, in several other countries; e.g. in Belgium, Canada, Denmark, Ireland, the Netherlands, New Zealand, Scotland and USA (Robert Pederson, the Danish Cancer Society, personal communication), but effect evaluations have not yet been published in scientific journals.

There are some limitations of the present study. The schools were randomly drawn from two different counties, but the schools receiving free fruit were for practical reasons randomly drawn from one of the two counties only. Some of the pupils (6th graders at nine schools in both counties, but no 7th graders) did also receive a FV educational programme during the intervention year (2001/2002). This educational programme showed, however, no effect in increasing FV intake [10,15], and all pupils in the FVMM cohort are therefore included in the present study to increase the statistical power. We also included the variable for the educational programme as a covariate in the model. However, the educational programme was highly correlated with class level and we found that using only class level as covariate gave a better fit (using the Akaike information criterion). We also performed analyses restricted to the 7th grade pupils, and the same conclusions apply although with this reduced sample we see a weaker significance for the FV variables (p-values were 0.02, 0.02, 0.04 for FV at school, FV all day and usual FV intake, respectively).

## Conclusion

The results show long-term effects of a free school fruit programme. The effects are in part mediated through higher participation in the national (paid) school fruit programme the years following the intervention.

## Competing interests

The author(s) declare that they have no competing interests.

## Authors' contributions

KIK conceived the study and is together with EB responsible for the design of the study. MBV, ØS and EB worked with the statistical models. ØS ran the final analyses. All authors contributed to the interpretation of the data. EB drafted the article, and MBV, ØS and KIK revised it critically. All authors have approved the final version of the manuscript.
